# Fatigue crack growth characteristics of Fe and Ni under cyclic loading using a quasi-continuum method

**DOI:** 10.3762/bjnano.9.93

**Published:** 2018-03-27

**Authors:** Ren-Zheng Qiu, Yi-Chen Lin, Te-Hua Fang

**Affiliations:** 1 Department of Mechanical Engineering, National Kaohsiung University of Science and Technology, Taiwan

**Keywords:** dislocations, fatigue crack growth, materials treatment effects, mechanics, shear stress

## Abstract

A quasi-continuum (QC) method based on the embedded atom method (EAM) potential was employed to investigate the fatigue crack growth and expansion characteristics of single-crystal Fe and Ni under cyclic loading modes I and II. In particular, the crack growth and expansion characteristics of Fe and Ni under cyclic loading were evaluated in terms of atomic stress fields and force–distance curves. The simulation results indicated that under cyclic loading, the initially damaged area of the crack will coalesce again after compression or shear to the initial geometry leading to a strengthening of the material. If no coalescence appears, the crack spreads rapidly and the material breaks. Moreover, under the cyclic loading of shear at any orientation, the slip dislocation observed in the materials considerably affects the release of stress.

## Introduction

When materials undergo cyclic loading, the growth of cracks in the material finally leads to fracture, which is referred to as fatigue. The fatigue behavior of structures is an important topic in fracture mechanics as fatigue failure is one of the major causes of accidents. Hence, it is imperative to investigate the fatigue cracking mechanism of nanocrystals for developing good, strong materials.

Considerable attention has been focused on the investigation of the fatigue crack growth behavior in single crystals under cyclic loading using molecular dynamics (MD), which is an effective tool for analyzing the mechanical deformation and mechanical properties of materials at the microscopic scale [1–13]. For face-centered cubic (FCC) metallic systems, Wu et al. [10] have investigated the fatigue crack growth in single-crystal Ni under different cyclic loading regimes. They found that different crack propagation and stress distributions lead to changes in fatigue crack growth rates and crack opening displacements. Ma et al. [11] have examined the effect of orientation on the fatigue crack propagation in single-crystal iron under cyclic loading, leading to differences in the crack growth rates and slip directions. The plastic deformation and double slip in single-crystal Ni and Cu under different loading orientations, including [111], [100], [110] and [101], have been reported by Potirniche et al. [12]. In addition, the fatigue crack growth of body-centered cubic (BCC) metallic systems under cyclic loading was analyzed by some MD simulations. Zhang et al. have examined the effect of the phase boundary on the fatigue crack propagation in an α/β-iron biphasic system under cyclic tensile loading [13]. They found that the nucleation of new cracks typically appears at the phase boundary because of dislocation pile-ups.

However, most of these studies using MD simulations have only focused on the fatigue fracture mechanisms. The relationship between the loading force and microstructure variation around the crack tip during the fatigue crack growth for BCC-Fe and FCC-Ni in different orientations has rarely been discussed simultaneously. In our previous study [14], the variation of the crack propagation in Fe and Ni during tensile processes with single orientation and different crack lengths and orientations is described. Hence, it is interesting to investigate the comparison of the crack growth behavior and slip system in Fe and Ni under a cyclic loading of tension or shear.

To completely analyze the crack growth and expansion characteristics of single-crystal Fe and Ni, the quasi-continuum (QC) method as proposed by Miller and Tadmor [15], instead of the traditional MD method, was employed as its computational efficiency is considerably better than that of the MD method for large-scale materials [16–22]. The results were discussed in terms of the atomic stress field and force–distance curves.

## Methodology

The QC method, which is a multiscale method that couples MD and the finite element method to analyze the mechanical characteristics of the material, was employed as the simulation method to examine the crack growth and expansion characteristics of single-crystal Fe and Ni under cyclic loading [19]. In the QC method, representative atoms are primarily used for calculation instead of all atoms, considerably decreasing the degrees of freedom and enhancing the simulation efficiency [14,19].

The energy calculation in the QC method is different from the other atomic calculation methods. In the system composed of *N* atoms without any external force, the total energy η(*u*) of the system can be expressed as follows [16]:

[1]
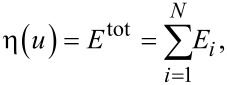


where *u* is the displacement vector; *E*^tot^ is the total energy of *N* atoms; and *E_i_* is the energy of atom *i.* The balance condition of the system is that by using the displacement vector *u*, the total energy is minimized. However, for the QC method, representative atoms are selected to approach the system composed of *N* atoms. Hence, the total energy is rewritten as [16]:

[2]
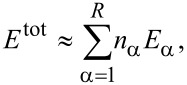


where *R* is the number of the representative atoms (*R* << *N*), *n*_α_ is the representative atom, and α is the number of the atoms.

The interaction between the atoms is described by the embedded atom method (EAM) potential. Dow and Baskes have proposed the EAM potential, which is a multibody potential [20–21]. In this study, the temperature used in the simulations was 0 K for all cases.

**Figure 1:**
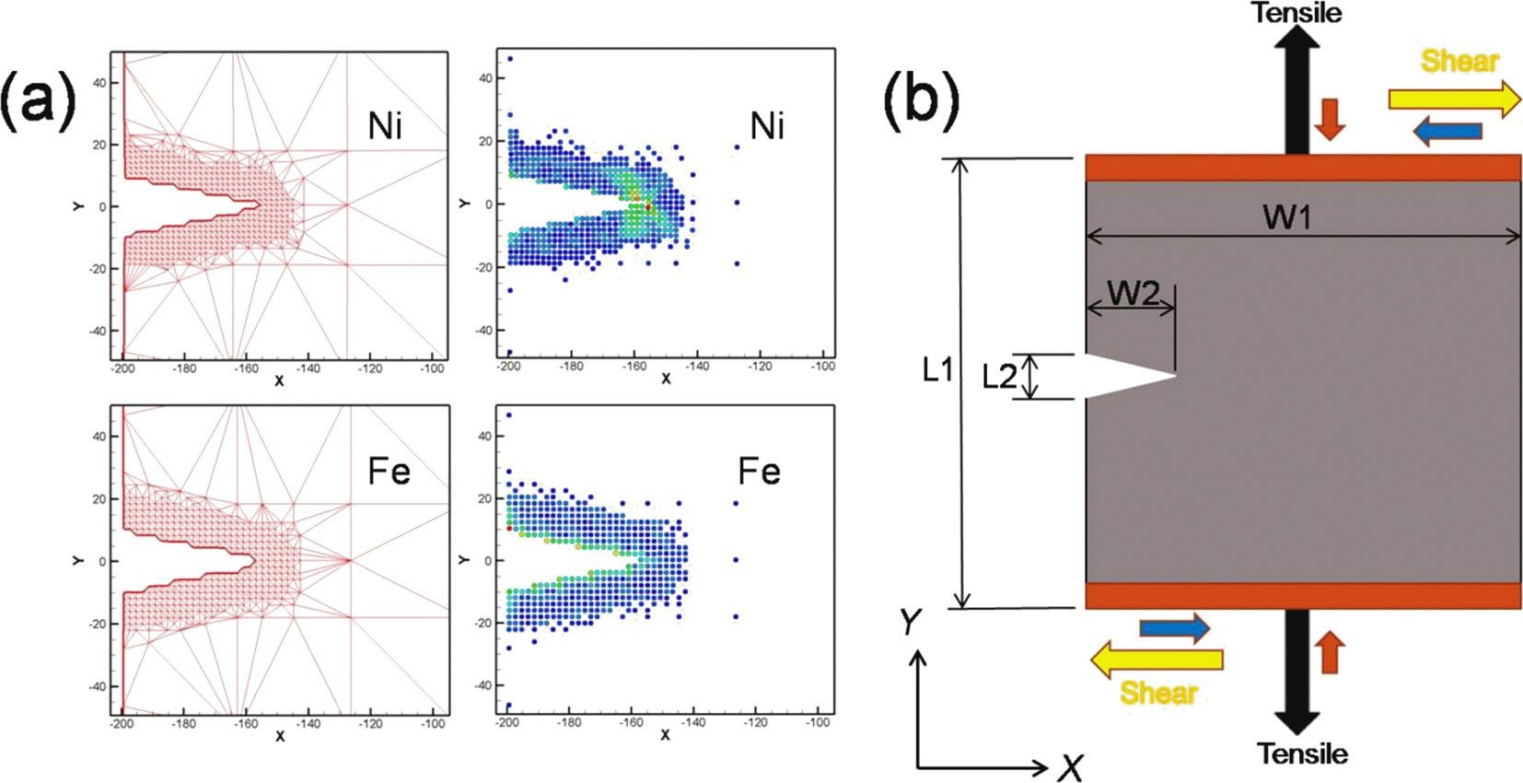
(a) Diagrams showing the physical models of Fe and Ni used in the simulations. (b) Diagram showing the sample under cyclic loading of tension–compression and shear–reverse shear, where W1 and L1 represent the width and length of the sample, which are 40 nm. W2 and L2 represent the width and length of crack of the sample, which are 1.76 nm and 4.4 nm, respectively.

Figure 1a shows the diagram of the physical model of Ni and Fe used in the simulations. The mesh refinement represents the areas of cracks that grow and expand during cyclic loading. To decrease the computational complexity, the other areas of the sample were treated with mesh coarsening. As the energy calculation at the interface between the continuum regions and the atomic regions is asymmetric, a so-called ghost force will be observed. However, Shenoy et al. [23] have proposed a correction method to fix the error originating from the ghost force in the solution. Figure 1b shows the sample under cyclic loading of tension–compression (mode I) and shear–reverse shear (mode II). W1 and L1 represent the width and length of the sample (40 nm). W2 and L2 represent the width and length of the cracks in the sample, which are 1.8 nm and 4.4 nm, respectively. The two layers of atoms located at the top and the bottom of the sample were fixed to support the entire system, with dimensions of approximately 40 nm × 1 nm in the *x*- and *y*-directions, respectively.

## Results and Discussion

### Crack growth and expansion characteristics under tension and compression

**Figure 2:**
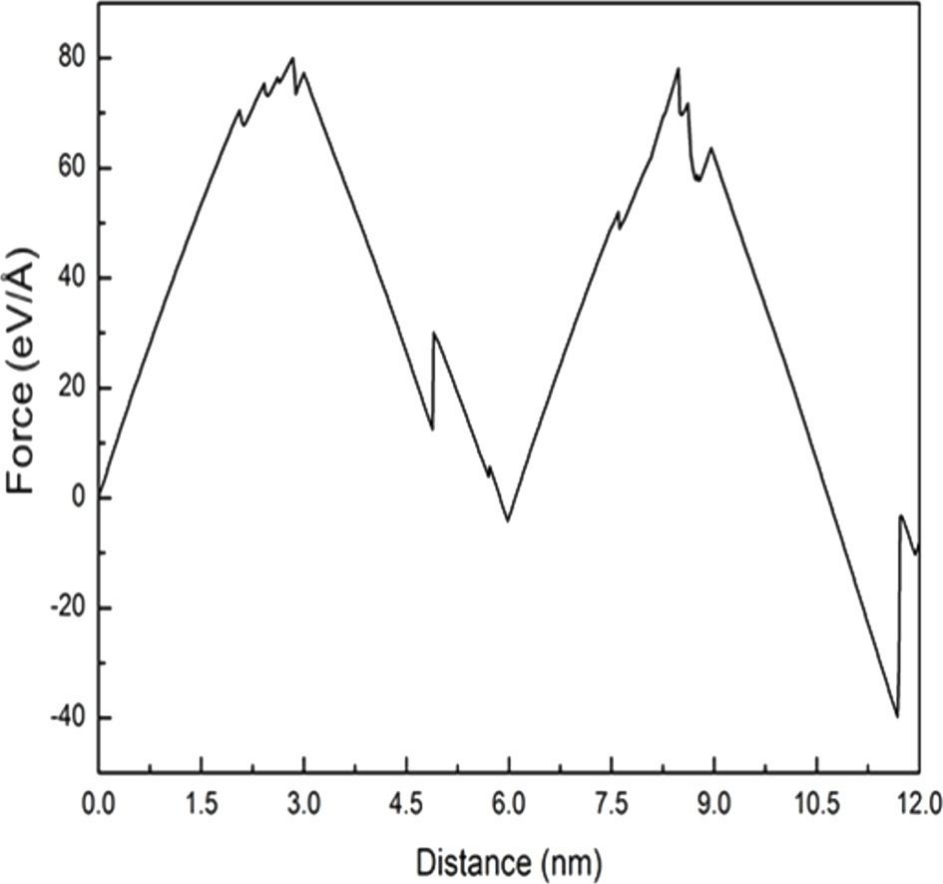
Force–distance curve of Ni under two times of cyclic loading, where the tension and compression distances of the sample are 3 nm.

The orientation for analyzing the crack growth characteristics under cyclic loading of tension and compression (mode I) was selected according to the similar atomic orientation between Ni and Fe [18], which is x-[100], y-[010], and z[001] for Ni and x-[110], y-[−110], and z[001] for Fe. Figure 2 shows the force–distance curve of Ni under two times of cyclic loading, where the tension and compression distances of the sample are 3 nm. Similar variations in the force occur for the first and second loading, with the maximum force being ca. 79 eV/Å.

**Figure 3:**
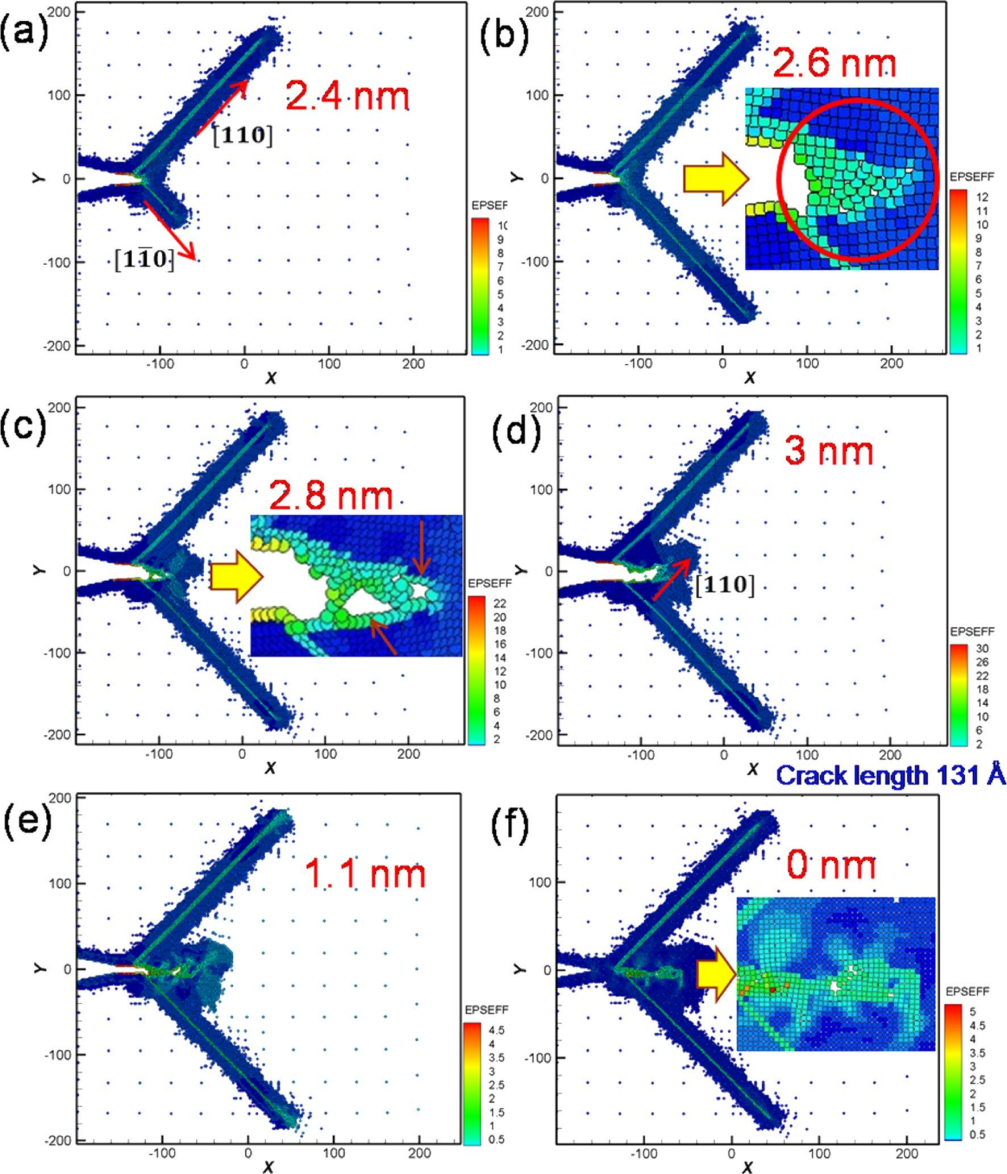
Crack growth and expansion diagrams of Ni under the first cyclic loading at different moving distances. The crack length is 131 Å at a tension distance of 3 nm.

**Figure 4:**
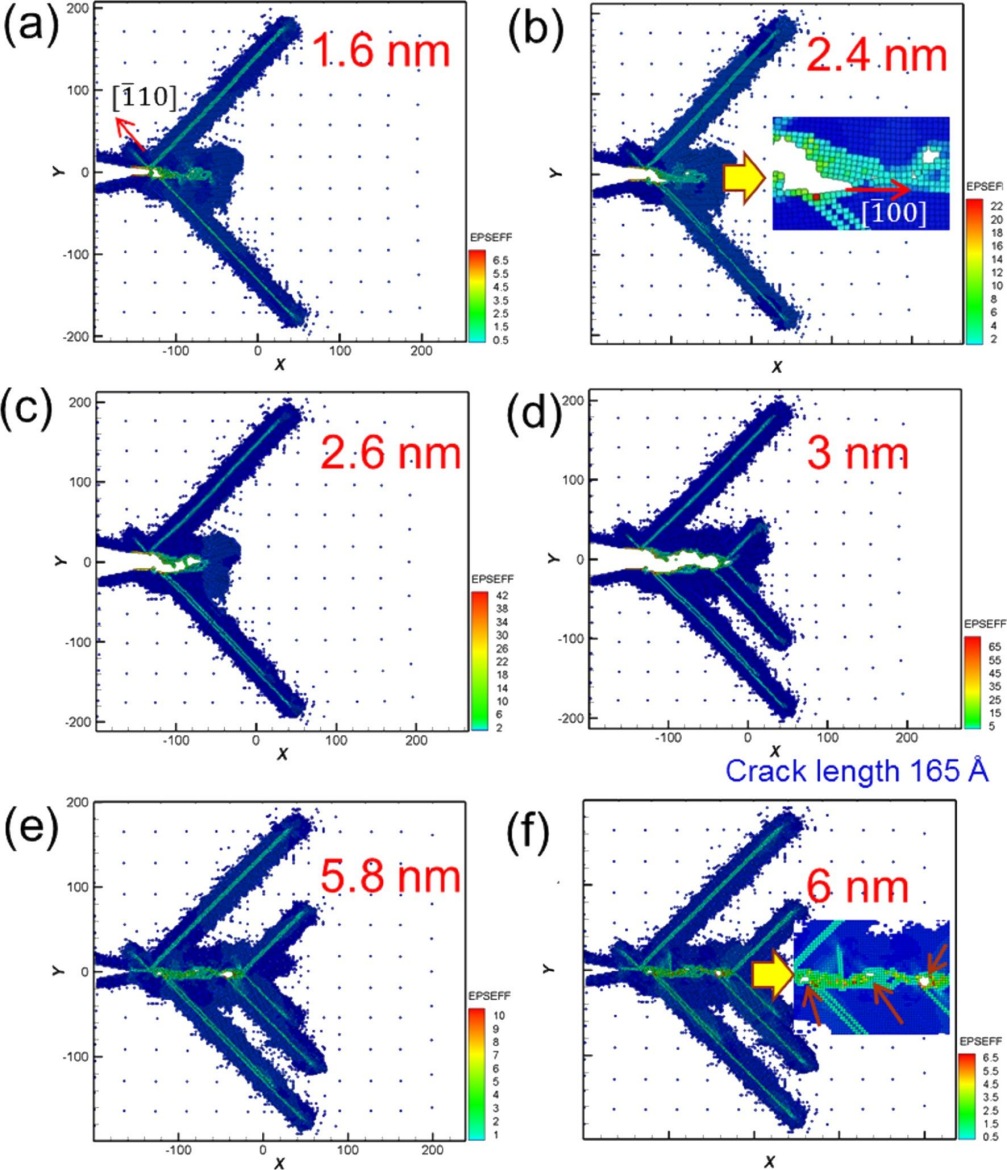
Crack growth and expansion diagrams of Ni during the second cyclic loading at different moving distances. The crack length is 165 Å at a tension distance of 3 nm.

The crack considerably affected the force output during cyclic loading. Figure 3 shows the microstructure evolution around the crack tip of Ni for different moving distances in the first cyclic loading. When the distance reached 2.4 nm, two slips were observed along the [110] and [1−10] orientations, leading to a split in the atomic stress field around the crack tip and the suppression of crack growth (Figure 3a). However, with the increase of tension, several small holes were observed around the crack tip (Figure 3b). These small holes combined into large holes, followed by the expansion of the crack tip toward the defect area (Figure 3c). Moreover, at a tension distance of 3 nm, where the crack length increased from 44 Å to 131 Å, the crack tip easily grew along the slip orientation of [110] [4,6] (Figure 3d). Next, the microstructure evolution around the crack tip for compression is shown in Figure 3e,f, assuming that the loading distance starts from 3 nm to 6 nm. The results indicated that the originally split parts of the sample coalesce, and defect holes are still present inside the crack tip even if the sample is compressed to the initial coordinates. That is, the sample cannot be restored to its original condition [4,10]. From the crack growth and expansion diagrams of the second cyclic loading shown in Figure 4, another [−110] slip orientation was observed when the tension distance reached 1.6 nm (Figure 4a). Under continuing tension, as can be observed in Figure 4b,c, the crack expanded along the [100] orientation, where small holes were observed in the sample during the first cyclic loading. At a tension distance of 3 nm, the crack tip became blunt, and the crack length increased to 165 Å.

The crack growth progressed under compression loading (Figure 4e,f). In Figure 4e, the initially split parts of the sample coalesce. This result is the same as that obtained for the first compression loading. When the sample is compressed to the initial coordinates, some holes are still present in the sample (Figure 4f). However, more and larger holes were observed in the sample under the second compression loading. In a previous study reported by Wu et al. [10], holes were observed in single-crystal Ni during the cyclic loading of tension and compression. Moreover, coalescence was observed during compression. These results are in good agreement with our results.

**Figure 5:**
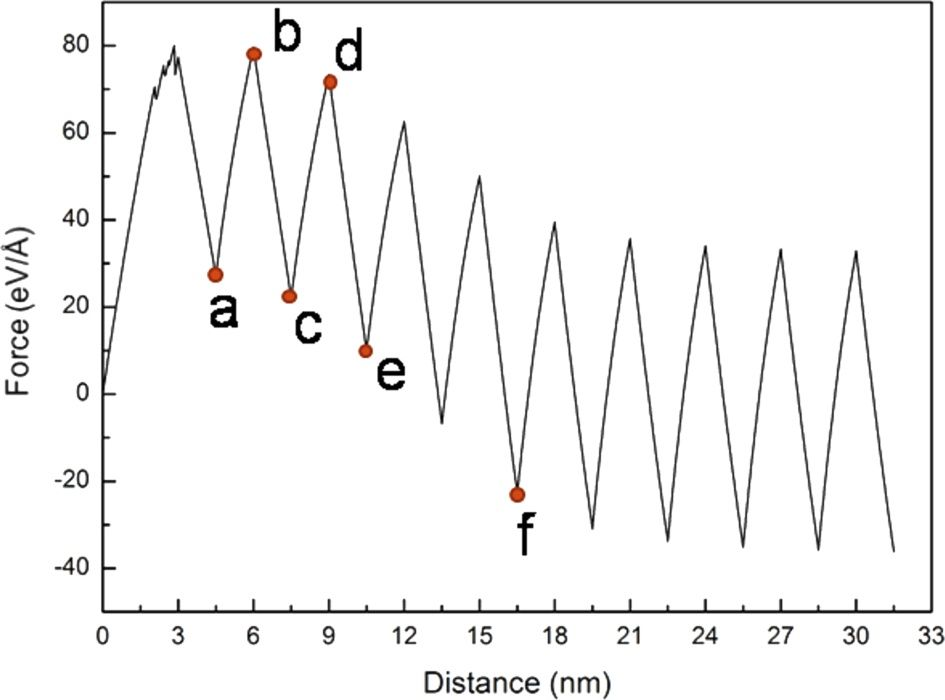
Force–distance curve of Ni under ten times of cyclic loading, where the first tension distance of the sample was 3 nm, and the subsequent compression and tension distances were 1.5 nm. The letters a–f represent the observation positions for the crack growth diagrams in Figure 6.

**Figure 6:**
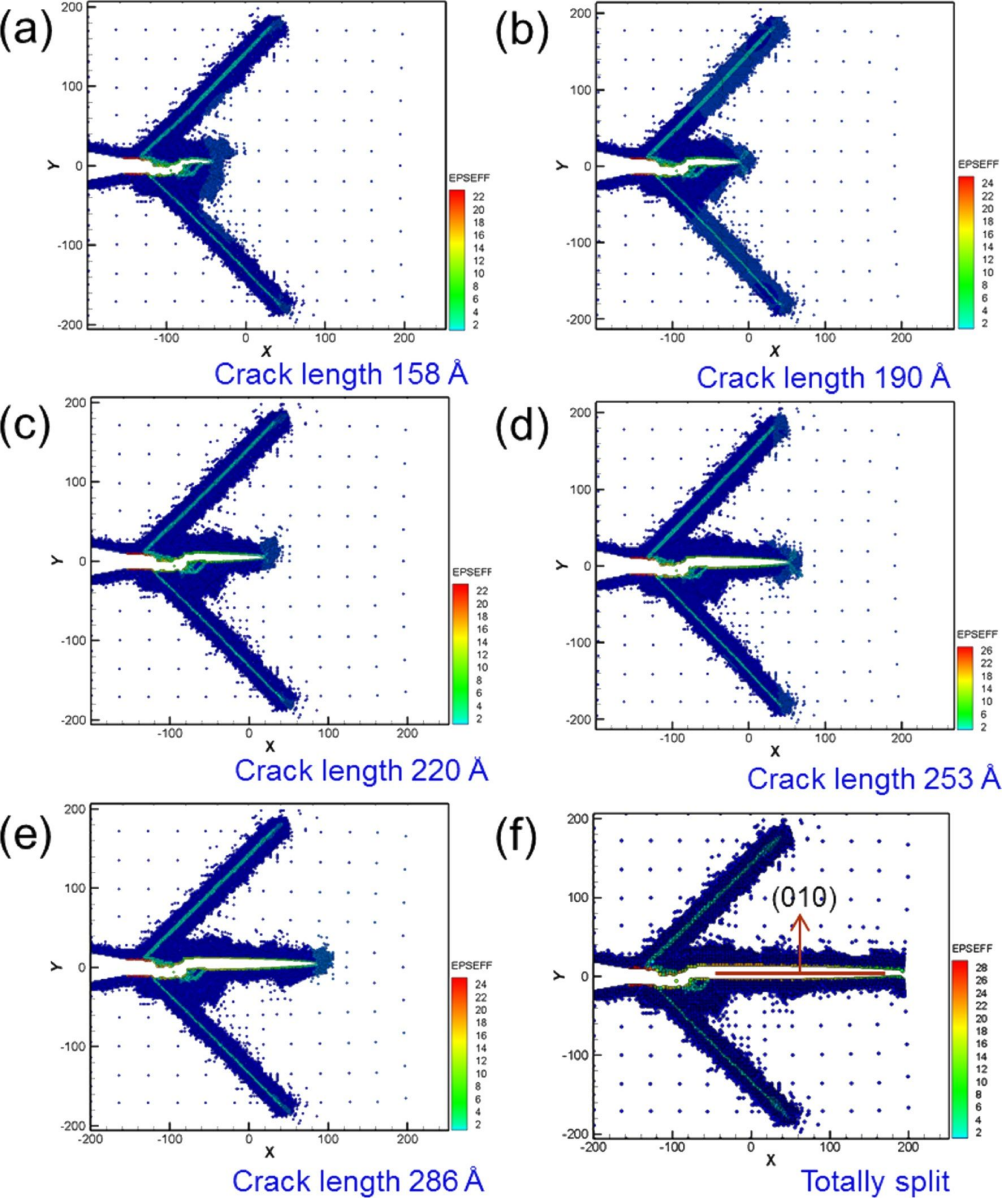
Crack growth and expansion diagrams of Ni corresponding to the observation positon a–f annotated in Figure 5.

To fully evaluate the microstructure evolution around the crack tip of the sample, the cyclic loading process was extended from two times to ten times. The force–distance curve of Ni under ten times of cyclic loading is shown in Figure 5 with a sample tension distance of the first cyclic loading of 3 nm and subsequent compression and tension distances of 1.5 nm. The letters a–f represent the observation position for the crack growth diagrams shown in Figure 6a–f.

From Figure 5, the maximum force appeared in the first cycle, and the variations in the force–distance curve tended to be stable after the fifth loading cycle. From the crack growth diagrams shown in Figure 6, each cyclic loading leads to a rapid fracture of the cracks inside the sample. Furthermore, the crack slightly expanded during compression. As the sample was not compressed to the initial position, coalescence was not observed, and the split occurred more rapidly. The sample completely split after the fifth cyclic loading (Figure 6f).

**Figure 7:**
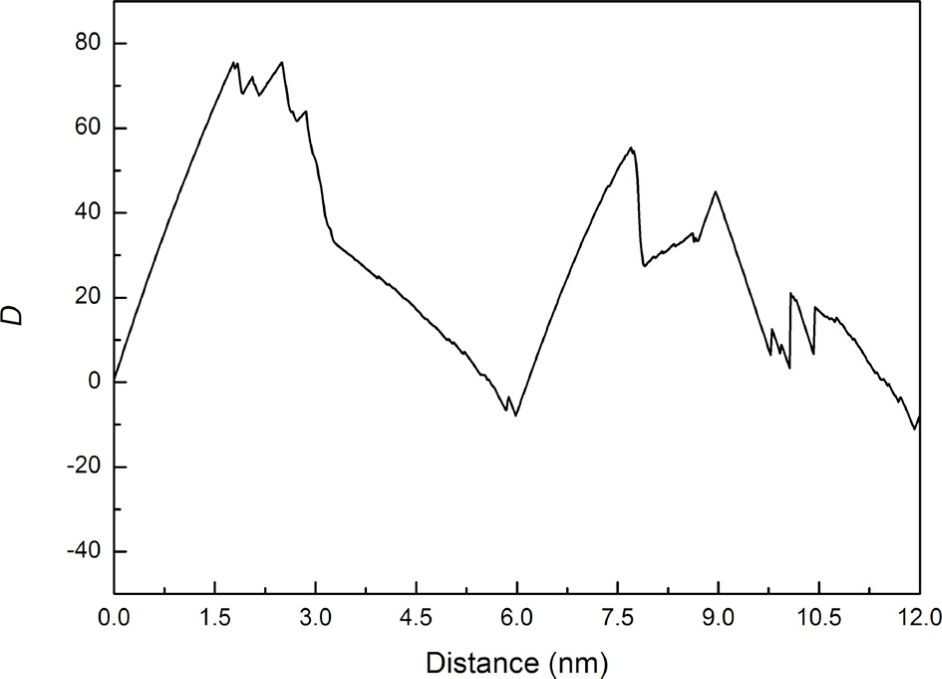
Force–distance curve of Fe under two times of cyclic loading, where the tension and compression distances of the sample are 3 nm.

**Figure 8:**
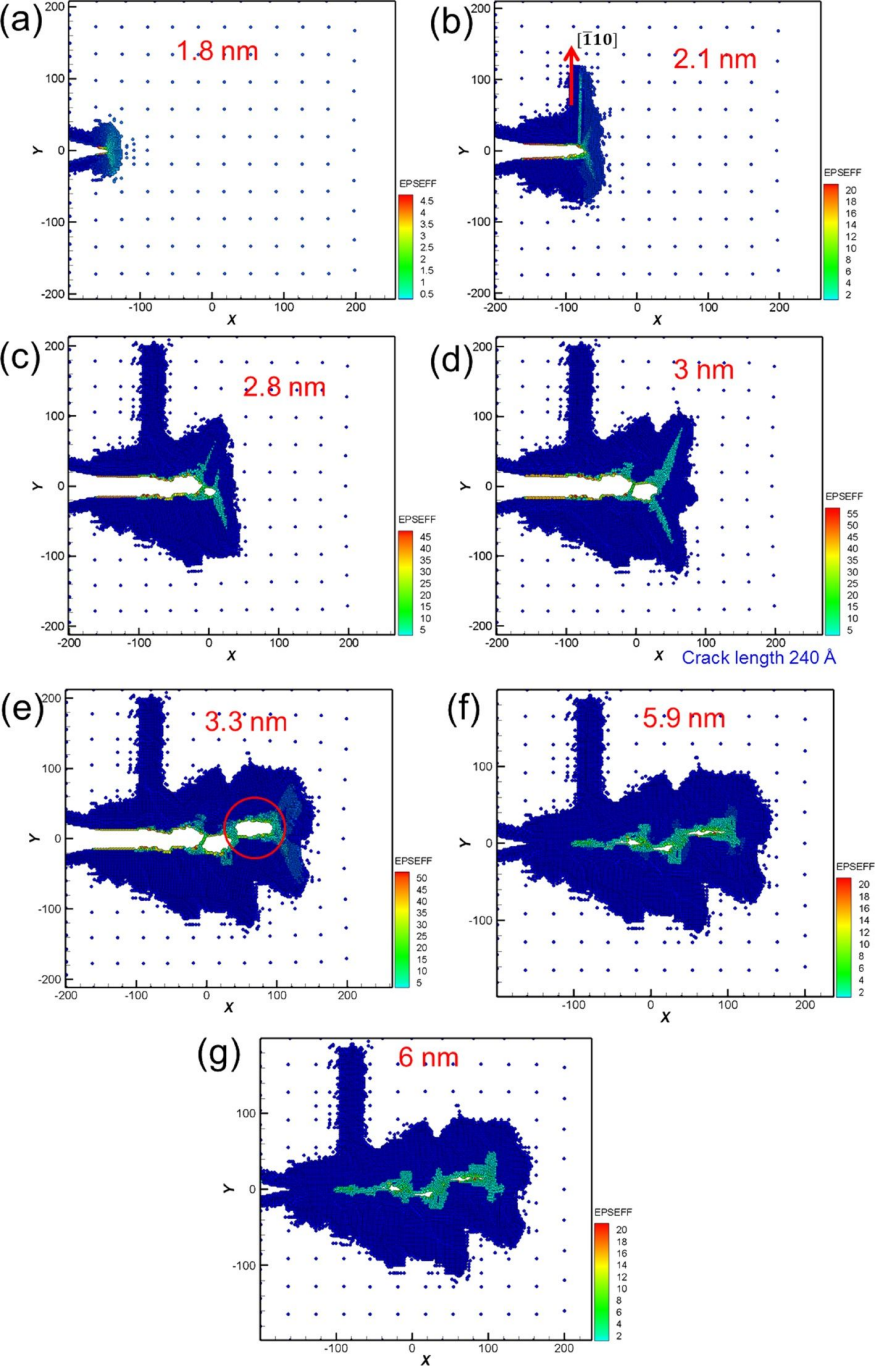
Crack growth and expansion diagrams of Fe under the first cyclic loading at different moving distances. The crack length is 240 Å at a tension distance of 3 nm.

The following paragraph will focus on the crack growth and expansion for single-crystal Fe under cyclic loading. Figure 7 shows the force–distance curve of Fe under two times of cyclic loading at sample tension and compression distances of 3 nm. Compared with the force–distance curve of Ni, the curve of Fe shows that the maximum force generated from the first tension loading is greater than that from the second tension loading. This result is different from that observed for Ni. In the microstructure evolution around the crack tip of Fe for the first cyclic loading, with increasing tension distance, a slip along the [−110] orientation was observed (Figure 8a,b). Similar to the case of Ni, the slip also caused the split of the atomic stress field around the crack tip and the suppression of the crack growth for Fe. This observation is in good agreement with that of Ma et al. [11]. Previously, Vatne et al. [18] have indicated that a blunting of the crack tip and the growth of the defect holes are observed during tension loading. This result is similar to that observed in Figure 8c. This crack growth variation of Fe was similar to that of Ni because of their similar atomic orientations. When the sample was under complete tension (Figure 8d), the growth of the hole was more obvious. In addition, the stress in the slip did not limit the growth of the cracks observed at the start of tension loading [22,24]. During compression loading (Figure 8e–g), the original small hole grew into the shape shown within the red circle of Figure 8e. As can be observed in Figure 8f, with increasing distance, the split parts shown in Figure 8e coalesced. However, the interior of the sample still retained defect holes. With the completion of the first cyclic loading (Figure 8g) the internal defect holes did not disappear but slightly decreased [7].

**Figure 9:**
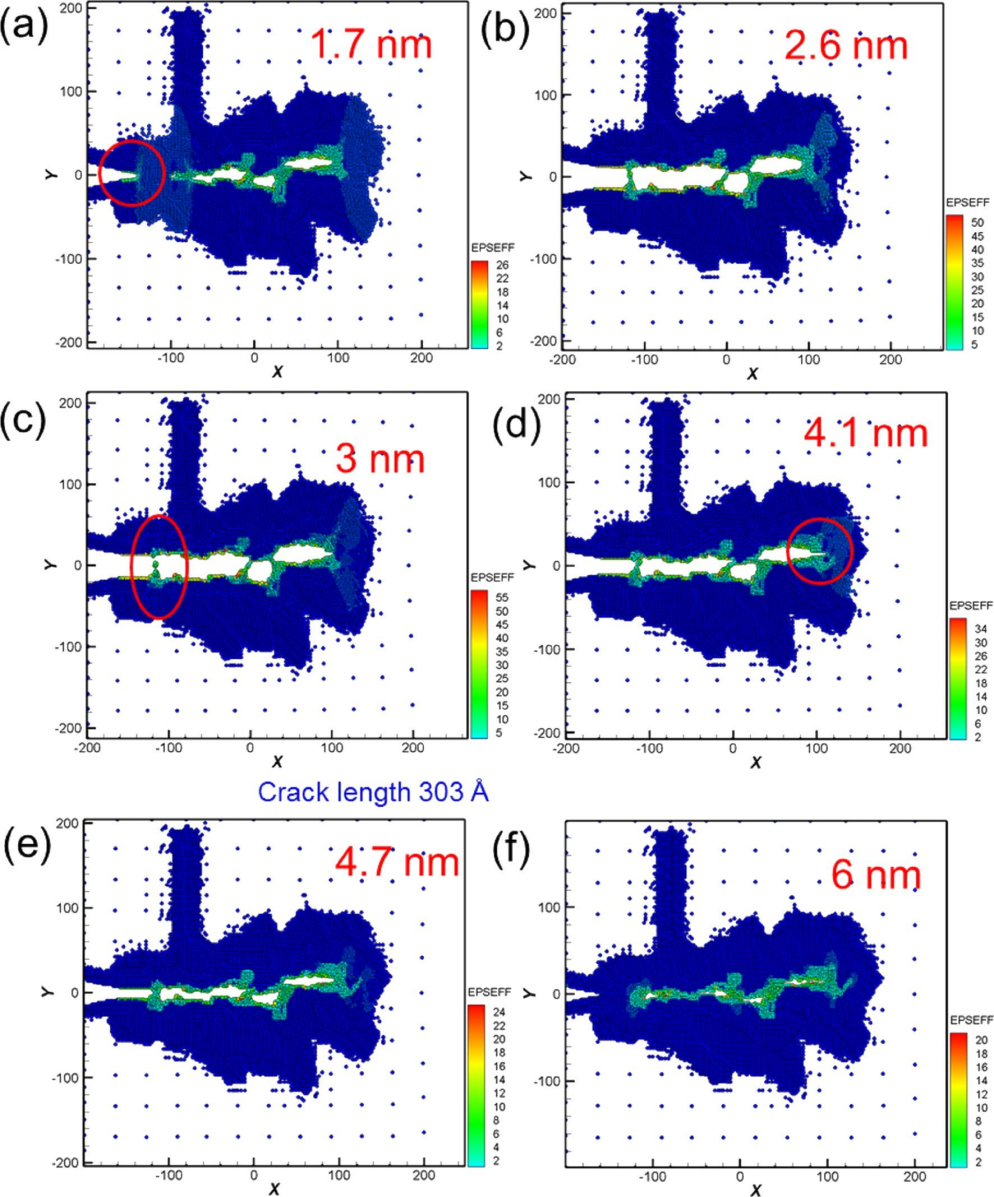
Crack growth and expansion diagrams of Fe under the second cyclic loading at different moving distances. The crack length is 303 Å at a tension distance of 3 nm.

Crack growth and expansion diagrams during tension in the second cyclic loading are shown in Figure 9a–c. During tension, the original internal defect grew and expanded. Moreover, the crack tip grew toward the orientation at which defects exist, as can be observed within the red circle shown in Figure 9a. This crack growth evolution was similar to that of Ni. At increasing distances of 2.6 and 3.0 nm (Figure 9b,c), the internal defects considerably increased, leading to structure fracture (red circle in Figure 9c). As mentioned above, the force generated from the second cyclic loading was less than that from the first cyclic loading (Figure 7). This was because when the first loading distance compressed to the initial coordinates, the number of the defect holes in single-crystal Fe was greater than that of Ni. Therefore, it is more facile to generate the crack expansion and fracture under the second tension loading [11]. During compression loading, the growth of the crack tip occurred, as can be observed by the red circle of Figure 9d. At a compression distance of 4.7 nm (Figure 9e), the crack tip coalesces. In addition, the bulge part of the internal structure is under compression. However, the defect holes were still observed even though the loading distance was compressed to the initial coordinates (Figure 9f).

**Figure 10:**
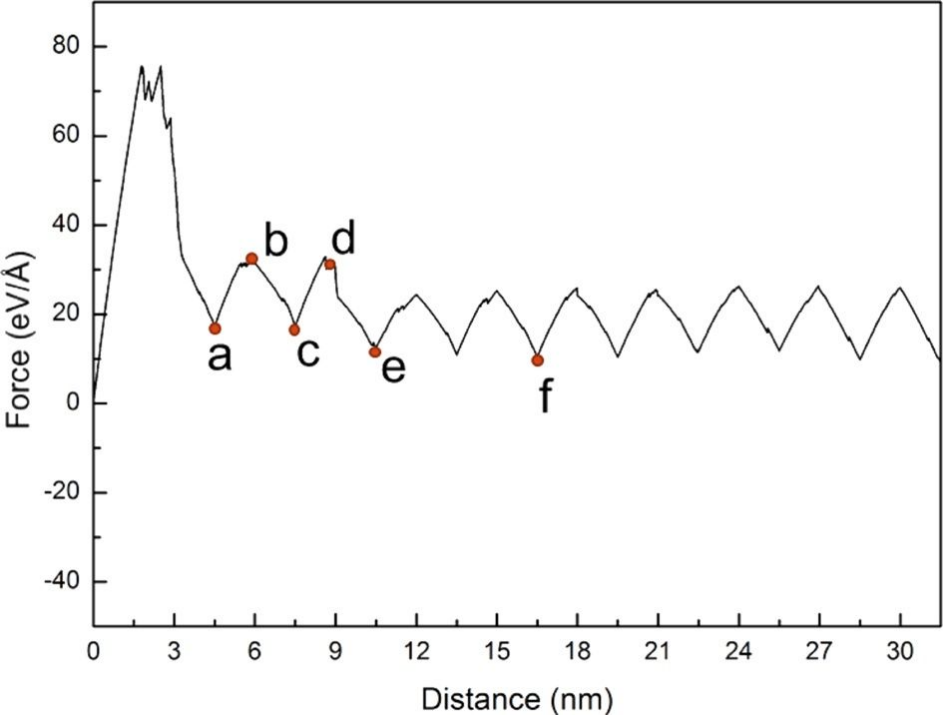
Force–distance curve of Fe under ten times of cyclic loading, where the first tension distance of the sample was 3 nm, and the compression and tension distances were 1.5 nm. The letters a–f represent the observation positions for the crack growth diagrams in Figure 11.

**Figure 11:**
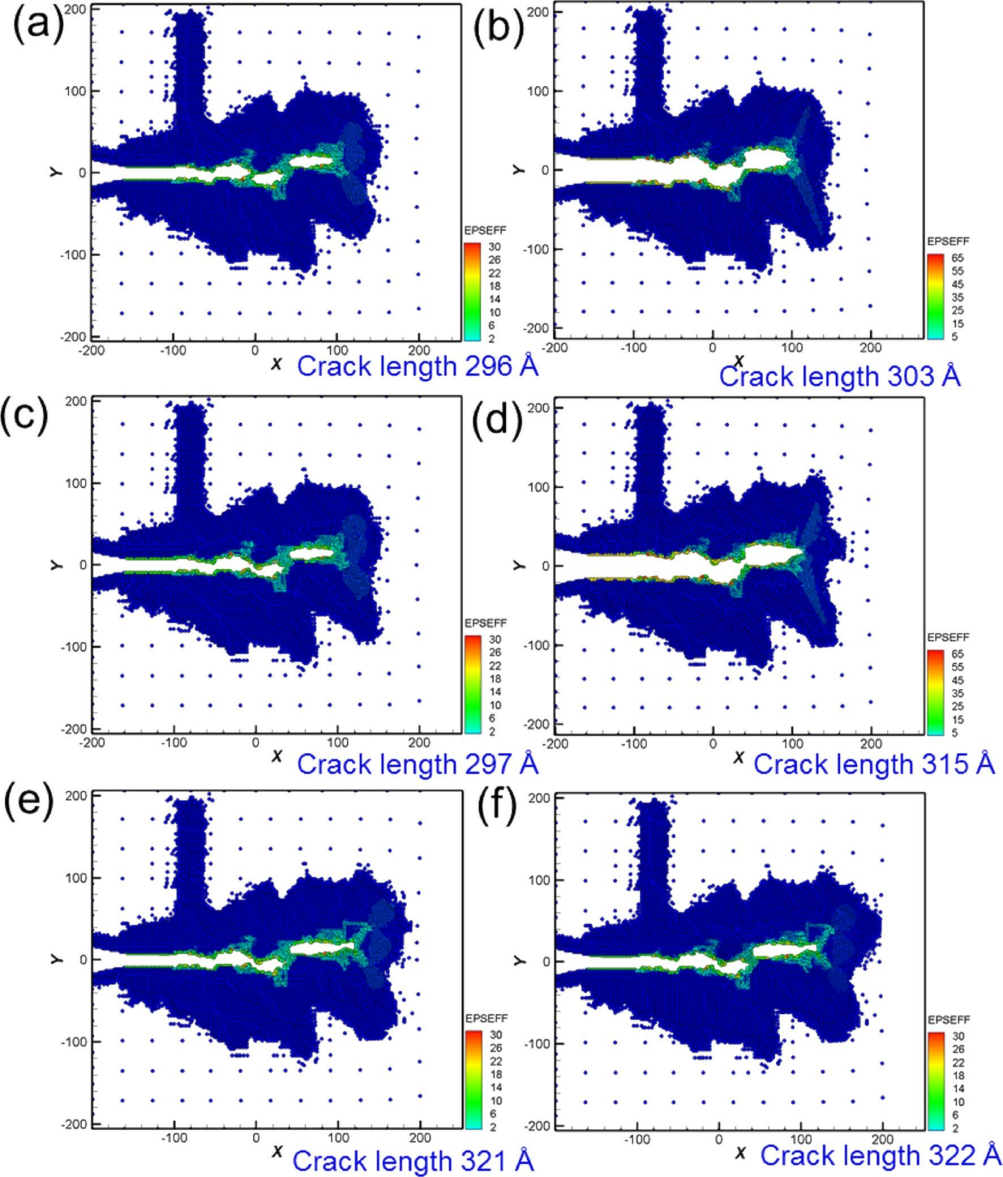
Crack growth and expansion diagrams of Fe corresponding to the observation positons a–f annotated in Figure 10.

Similarly, the force–distance curve of Fe, the number of cyclic loadings was extended from two to ten, with a sample tension distance of 3 nm and compression and tension distances of 1.5 nm (Figure 10). The maximum force was observed during the first cyclic loading. Moreover, the variations of the force–distance curve of Fe tended to stabilize after the third cyclic loading. Figure 11 shows the crack growth and expansion diagrams of Fe corresponding to the observation positions a–f shown in Figure 10. Unlike the Ni sample, the Fe sample did not split completely after the fifth cyclic loading (Figure 10f). Instead, coalescence of the internal crack of single-crystal Fe was observed during the cyclic loading process, which restrained the growth and expansion of the crack tip. The expansion of single-crystal Ni was rapid because the internal cracks did not coalesce.

**Figure 12:**
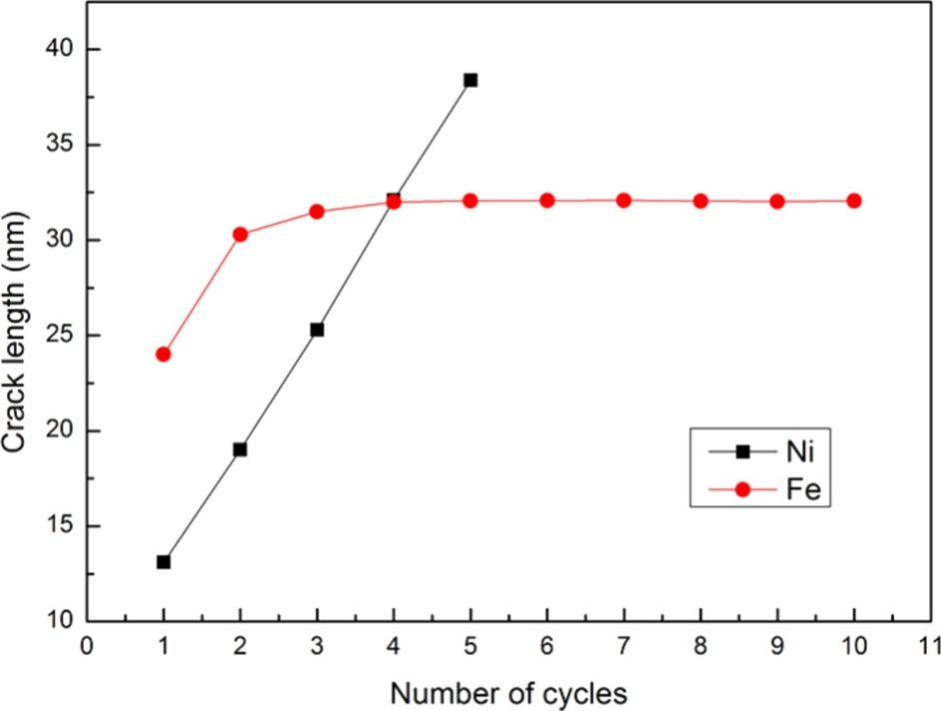
Comparison between the growth of crack length for Ni and Fe under ten times of cyclic loading.

A comparison of growth and crack length for Ni and Fe under ten times of cyclic loading is shown in Figure 12. The Ni sample split after the fifth cyclic loading, while the crack length of Fe remained almost constant after the fourth cyclic loading because of the conjugation of the cracks.

### Crack growth and expansion characteristics under shear and reverse shear

**Figure 13:**
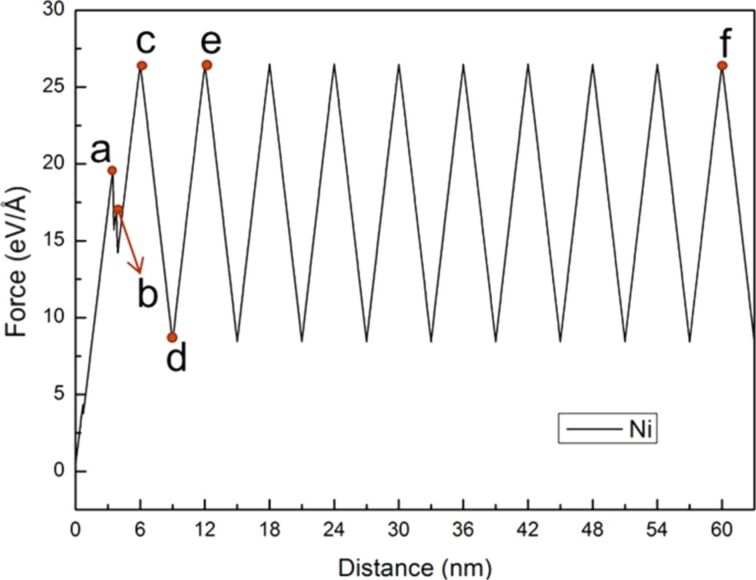
Force–distance curve of Ni under ten times of cyclic loading, where the first shear distance of the sample was 6 nm, and the reverse shear and shear distance were 3 nm. The letters a–f represent the observation positions for the crack growth diagrams.

In this section, the crack growth and expansion characteristics under cyclic loading of shear and reverse shear (mode II) for FCC-Ni and BCC-Fe was analyzed. For mode II, the same model as shown in Figure 1b was employed, where W2 and L2 were set as 1.76 nm and 20 nm, respectively. Orientation I was selected for the analysis for Ni and Fe, which was set to be x-[110], y-[−110], and z[001]. Figure 13 shows the force–distance curve of Ni under ten times of cyclic loading at a sample shear distance during the first cyclic loading of 6 nm and reverse shear and shear distances of 3 nm. The letters a–f represent the observation position for the growth of crack length.

**Figure 14:**
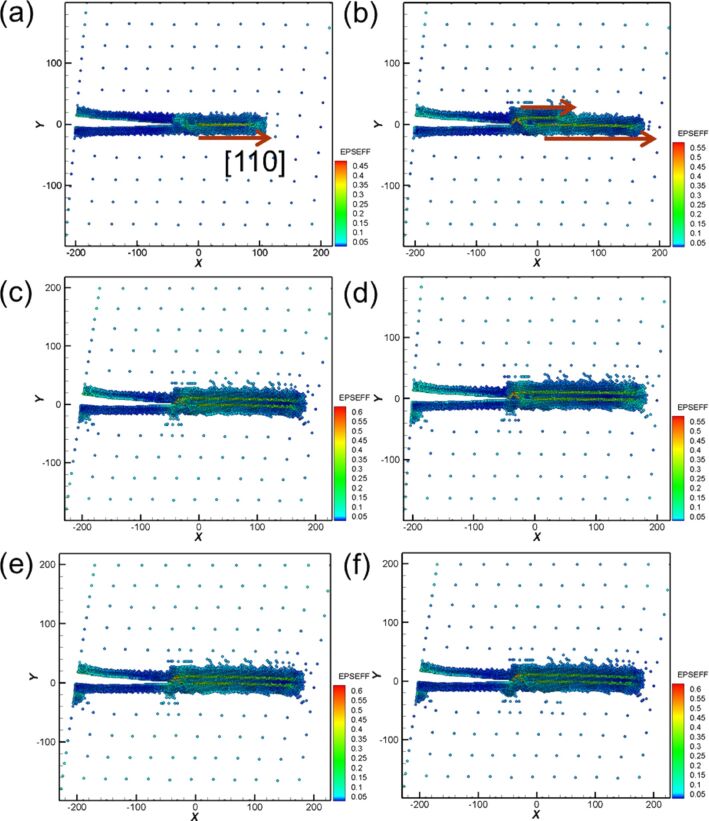
Crack growth and expansion diagrams of Ni corresponding to the observation positions a–f in Figure 13.

After the first cyclic loading, the variations of the curve became steady. When the amount of shear deformation reached a specific point as shown in Figure 14a, a slip along the [110] orientation generated from the area of the crack tip was observed, which released the internal stress. Moreover, with increasing shear distance to the point b shown in Figure 14b, another slip was generated along the [110] orientation. The length of the second slip dislocation extended to the same length as the first one. When the first shear distance of the sample reached 6 nm (Figure 14c), the crack tip did not expand in the same manner as that observed under the cyclic loading process of mode I. Instead, the crack tip only generated dislocations, and the position of the opening was slightly pulled up. For the reverse shear process during the first cyclic loading, as can be clearly observed from Figure 14d, the original slip dislocation observed in the sample during the shear still existed rather than returning back to the initial state, indicating that the interior of the sample is already damaged. Moreover, no slips were observed in the sample during the reverse shear process. Figure 14d–f shows the crack growth and expansion diagrams under the second and tenth shear process. The microstructure evolution was very similar. This result corresponded to the force–distance curve of Ni shown in Figure 13, indicating that the variations of the curve tends to be steady after the first cyclic loading process.

**Figure 15:**
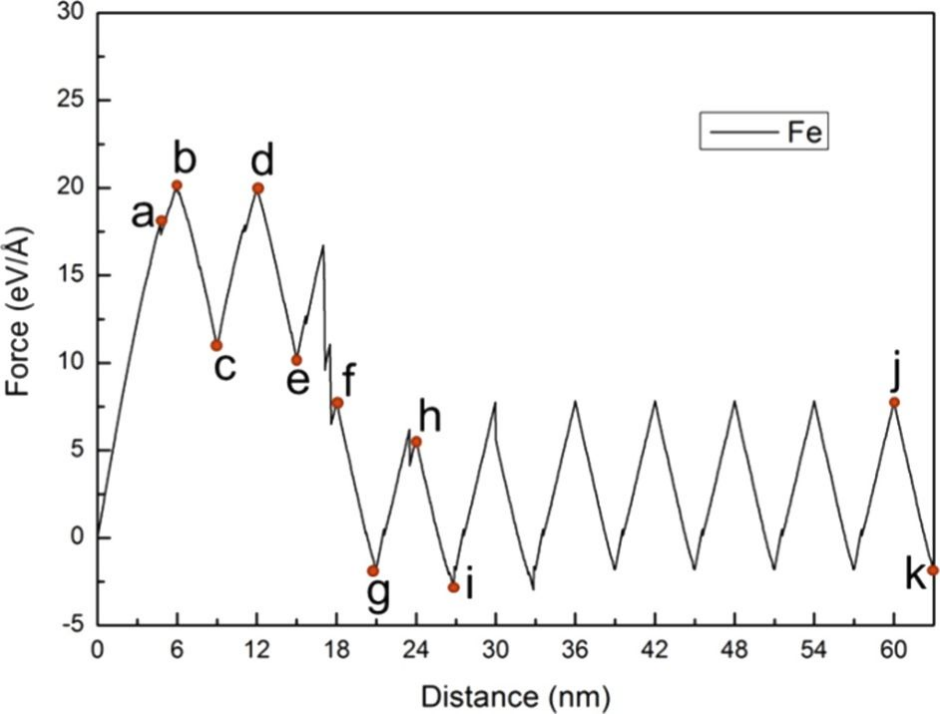
Force–distance curve of Fe under ten times of cyclic loading, where the first shear distance of the sample was 6 nm, and the reverse shear and shear distance were 3nm. The letters a–k represent the observation positions in Figure 16.

Next, the relationship between the force–distance curve and crack growth and expansion diagrams for Fe under ten times of cyclic loading of shear and reverse shear processes in the same orientation I was discussed. The force–distance curve of Fe tended to be steady after the fourth cyclic loading (Figure 15).

**Figure 16:**
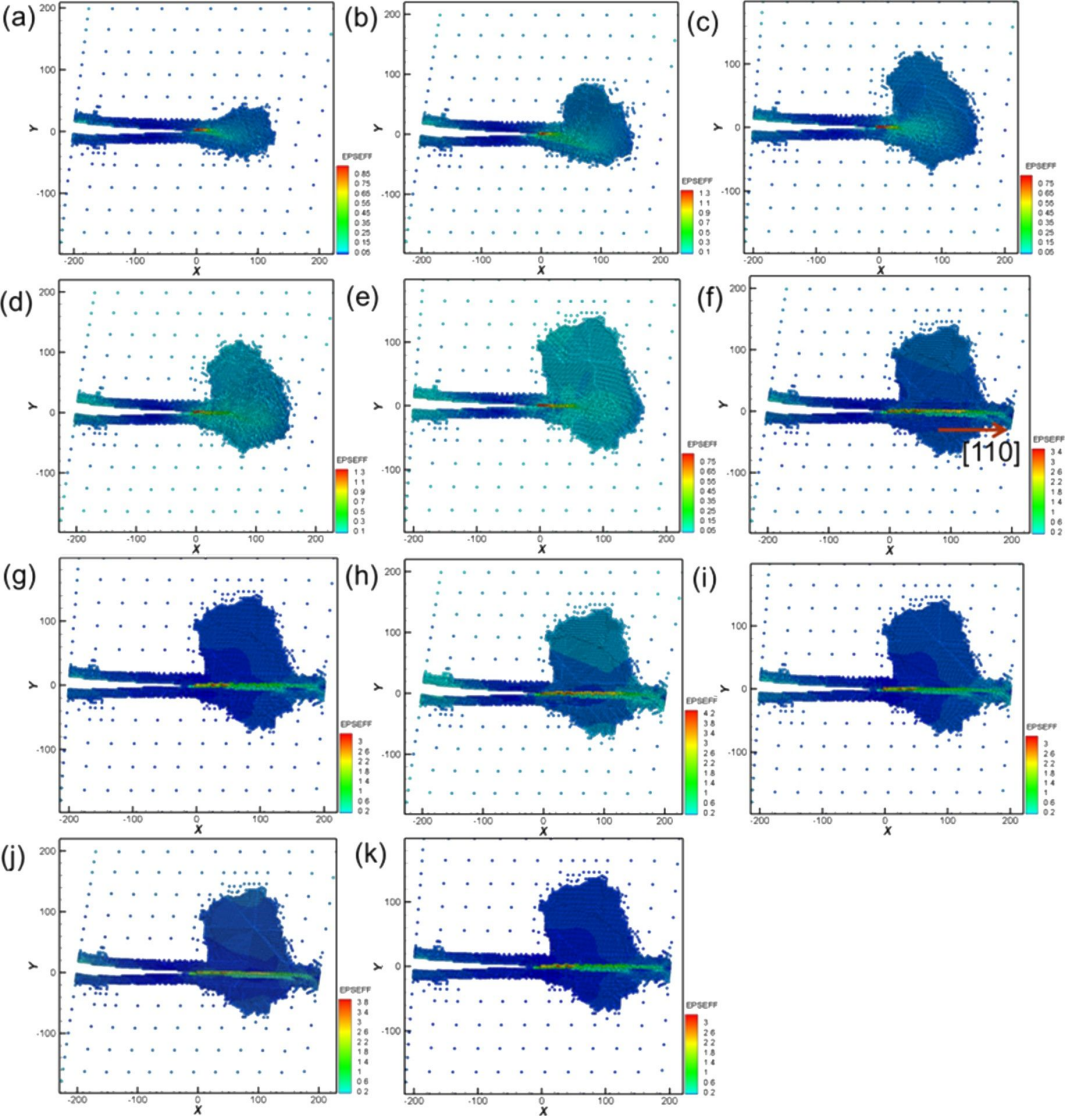
Crack growth and expansion diagrams of Fe corresponding to the observation positons a–f annotated in Figure 15.

During the first cyclic loading (Figure 16a–c), Figure 16a shows that mixed dislocations extended from the crack tip to the internal structure. This observation is different from the case of Ni generating regular and clear slip orientations. With the increasing amount of shear (Figure 16b) the dislocations expanded. In the reverse shear process of the first cyclic loading (Figure 16c), the area of the mixed dislocations expanded again because of the effect of the reverse shear. For the following cyclic loading processes, the crack growth and expansion diagrams are shown in Figure 16d–k. In the second loading process shown in Figure 16d,e, the internal mixed dislocations expanded again, and the affected area also increased. In particular, in the third loading process shown in Figure 16f, a slip with [110] orientation was observed, which directly extended to the boundary of the structure. This slip restrained the growth of the original internal mixed dislocations, leading to the steady microstructure evolution during the fourth to the tenth cyclic loading. The force of the first and second loading processes was greater than that of the other cyclic loading process because the mixed dislocations inhibited the release of the internal stress. The force did not start to decrease until the appearance of the slip in the third loading process. In our previous study [14], we showed that the stress varied with different crack lengths and the orientation plays an important role for the materials, which is in good agreement with the discussion above.

### Crack growth and expansion characteristics under shear and reverse shear along different orientations

**Figure 17:**
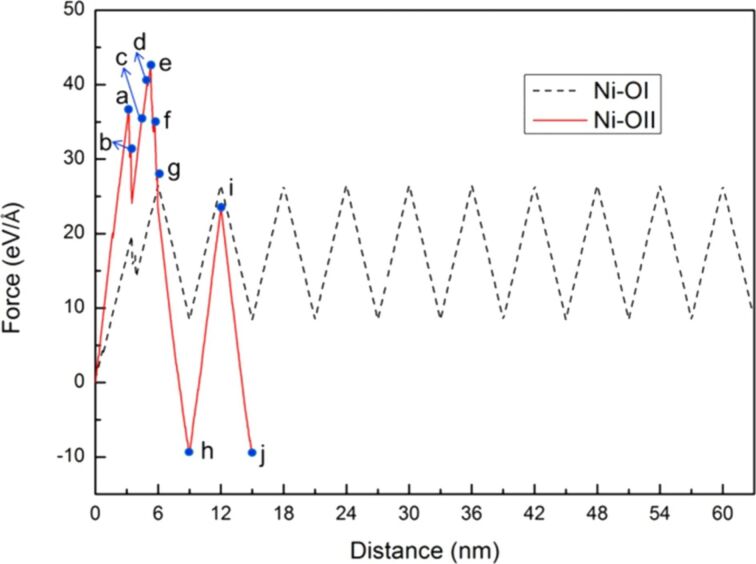
Force–distance curve of Ni along orientation I (black dashed line) and orientation II (red line) under ten times of cyclic loading, where the first shear distance of the sample was 6 nm, and the reverse shear and shear distance were 3nm. The letters a–j represent the observation positions in Figure 18.

In this section, the comparison of crack growth and expansion characteristics between single-crystal Ni and Fe under cyclic loading mode II for the different orientations is further discussed [17,25] because of the dependence of the microstructure variation of the sample on the orientation. Two orientations (orientation I (x-[110], y-[−110], and zz[001]) and orientation II (x-[100], y-[010], and z[001]), were selected for discussion. Figure 17 shows the comparison between the force–distance curve of Ni in along orientations I and II under ten times of cyclic loading. The force experienced along orientation II was clearly much greater than that along orientation I.

**Figure 18:**
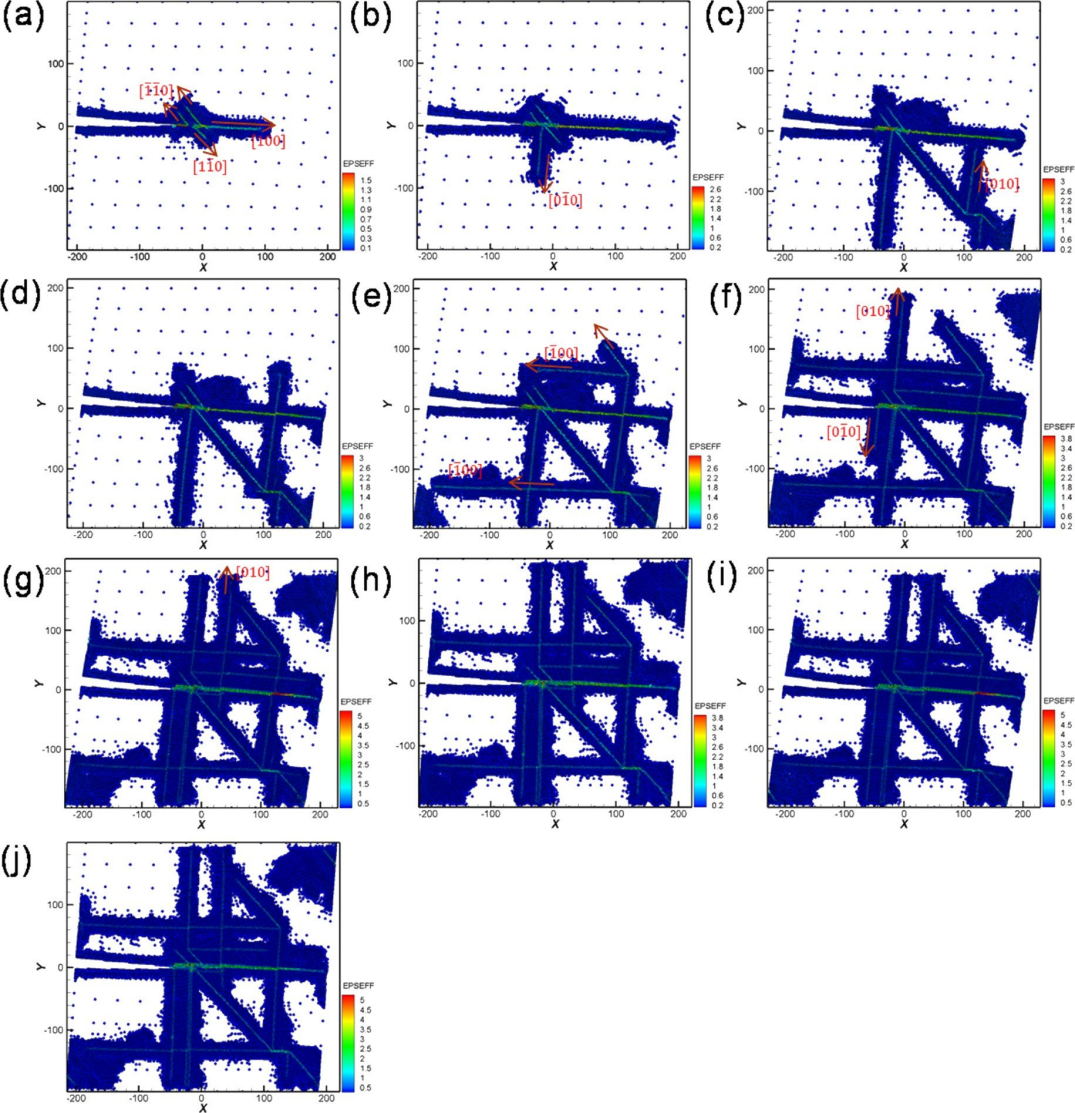
Crack growth and expansion diagrams of Ni along orientation II corresponding to the observation positons a–j in Figure 17.

During shear loading, dislocations along the [100] orientation were generated from the crack tip (Figure 18a). Moreover, slip dislocations along the [110] and [−1−10] orientations (Figure 18a–j) indicate the orientations that permit easy slipping in a slip system of FCC. With increasing shear loading distance, a slip dislocation was observed along the [1−10] orientation around the crack tip. Furthermore, the initial dislocation along the [100] orientation extended to the boundary of the sample. Another dislocation was observed along the [010] orientation in the sample, and the extension area of the initial dislocations increased with increasing loading distance (Figure 18c). All of the dislocations continued to extend to the boundary of the sample, leading to a rapid fracture of the material (Figure 18d–g). In addition, this result indicated the force that the sample can experience rapidly decreases (Figure 17). In the reverse shear process during the first cyclic loading (Figure 18h), the initial dislocations did not reappear. In the second cyclic loading shown in Figure 18i,j, as the internal sample of the material was severely broken during the first cyclic loading, the microstructure evolution did not change considerably. Moreover, the cyclic loading for Ni in orientation II could only be carried out twice because the fracture caused by the dislocations already affects the setting boundaries considerably.

**Figure 19:**
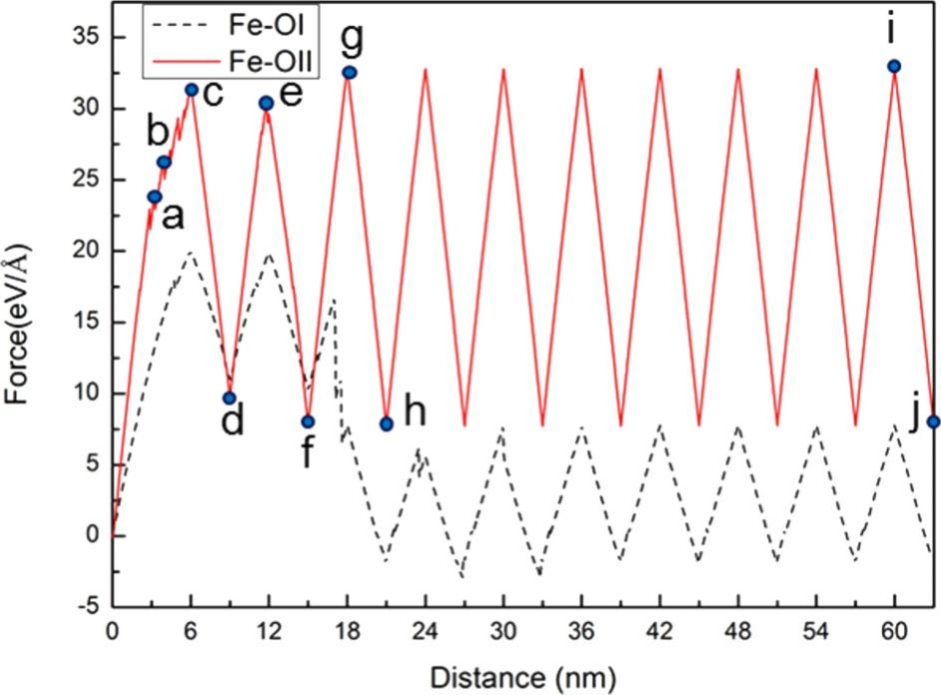
Force–distance curves of Fe along the orientation I (black dashed line) and orientation II (red line) under ten times of cyclic loading, where the first shear distance of the sample was 6 nm, and the reverse shear and shear distance were 3nm. The letters a–j represent the observation positions in Figure 20.

In Figure 19, BCC-Fe is discussed. The force experienced along orientation II was considerably greater than that along orientation I. This result is similar to that observed for Ni. Moreover, the variations in the force–distance curve of Fe tended to be steady after the second cyclic loading.

**Figure 20:**
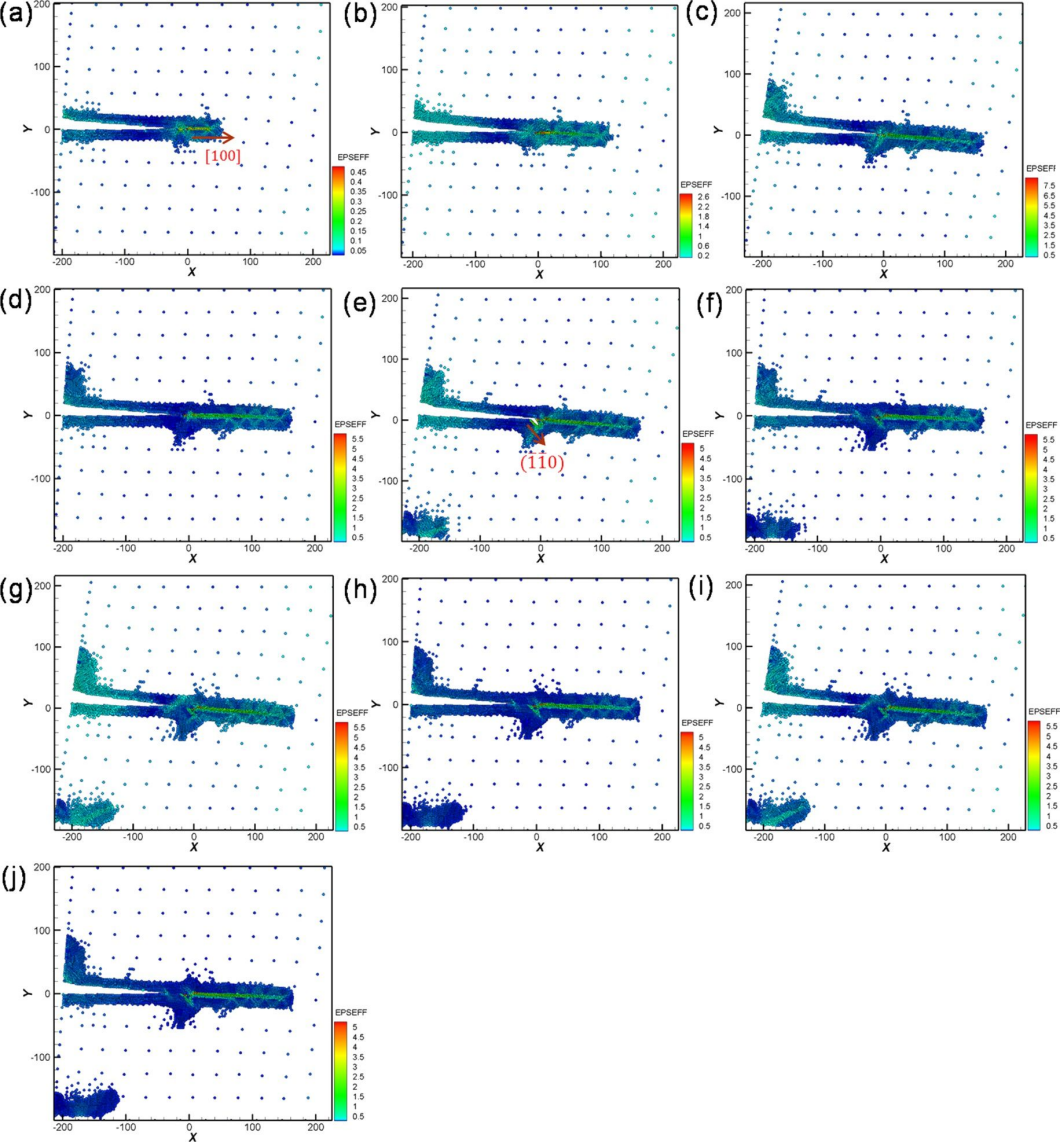
Crack growth and expansion diagrams of Fe along the orientation II corresponding to the observation positons a–j in Figure 19.

Figure 20 shows the crack growth and expansion diagrams along orientation II corresponding to the observation positions a–j shown in Figure 19. With the increase in the shear loading distance to point a, as shown in Figure 20a, a slip along the [100] orientation from the crack tip was observed. With the increase in the shear loading distance to point b, the expansion of the original slip continued (Figure 20b). Moreover, the upper position of the opening of the crack tip was pulled up because of the effect of the shear process, which is more obvious in Figure 20c. However, for the reverse shear process during the first cyclic loading shown in Figure 20d, the variation in the crack growth was not considerable [26]. For the second cyclic loading, the crack tip was slightly split and expanded along the [−1−10] direction (Figure 20e). Moreover, the destruction of the boundary of the left corner of the sample began. The maximum force in the second cyclic loading process was less than that in the first cyclic loading process because of the splitting of the crack tip. In the reverse shear process during the second cyclic loading, the original split area of the crack tip coalesced. However, no splitting was observed for the sample from the third to the tenth cyclic loading process (Figure 20g–j). Moreover, the maximum force of the third cyclic loading almost recovered to the same value of the maximum force of the first cyclic loading (Figure 19). The slip dislocation observed at the start or later affects the output of the maximum force. In addition, the maximum force is affected by the fact that the crack is split [27–29]. Our results are similar to those reported previously [26], indicating that dislocations from the crack tip along different orientations are also different. Moreover, the fatigue strength also depends on the orientation.

## Conclusion

The fatigue crack growth and expansion characteristics of single-crystal Fe and Ni under the cyclic loading modes I and II were examined by a QC method. The following conclusions were obtained: (1) Under cyclic loading of tension and compression, if coalescence of cracks is observed during compression, the expansion of the crack will be restrained. Some small defect holes inside the materials are observed even in the presence of coalescence. If there is no coalescence, the material will be rapidly fractured. Moreover, the crack slightly grows during compression. (2) For a similar atomic orientation of Ni and Fe, the Ni sample will be rapidly fractured after less than ten times of cyclic loading, because there is no coalescence phenomenon in the Ni sample. (3) Under cyclic loading of shear and reverse shear in the same orientation, the variations of the force–distance curve become steady after a certain number of cycles. Moreover, the order of priority of the dislocations generated from the crack tip affects the release of stress inside the material. (4) Under cyclic loading mode II along different orientations, the force experienced along orientation II is considerably greater than that along orientation I for Fe and Ni. In Fe, the appearance of the slip dislocations and the steadying of the variations in the force–distance curve is related to the number of the cycles.
